# Supplementary material for: Physiology can predict animal activity, exploration, and dispersal
Source: Commun Biol. 2022 Feb 3;5:109. doi: 10.1038/s42003-022-03055-y (PMC8814174; doi:10.1038/s42003-022-03055-y)
Supplement: Supplementary file 2 — Supplementary Information [file 42003_2022_3055_MOESM2_ESM.pdf]

SUPPLEMENTARY INFORMATION

Physiology can influence animal activity, exploration, and dispersal

Nicholas C. Wu, & Frank Seebacher

School of Life and Environmental Sciences, The University of Sydney, NSW 2006, Australia

Supplementary Methods

Data exclusion criteria

- Non-English literature, books without links to primary source, and non-peer-reviewed articles.
- Passive dispersal such as human transportation of wildlife from native to new environments, parasite dispersal, and abiotic driven dispersal. In addition, dispersal metrics related to offspring success such as the production of dispersing gametes/eggs or the colonisation success of offspring from water/air currents.
- Categorised dispersal polymorphism. i.e., winged and non-winged insects (Zera and Denno, 1997).
- Migration as defined by Nathan et al. (2008).
- Studies that did not define specific metabolic rate measurement.
- Response variables measured at test temperatures different from acclimation temperatures (e.g. animals acclimated at warm treatment but response measured at an acute cold treatment).
- Interspecific correlation studies of physiology and movement.
- Experimental manipulations that confound comparisons of movement, such as food restriction, exposure to pollutants.
- Molecular responses not directly related to physiological traits such as mRNA expression or transcriptomics.
- Principal component variables where body condition was grouped with other morphological variables.
- Artificial selection lines for more active, more explorative individuals.
- Within-individual correlation studies which reflect repeatability of measurements.

Conversion statistics

The correlation coefficient (r) was calculated from inferential statistics with the following equations from Lipsey and Wilson (2001), Nakagawa and Cuthill (2007), and Noble et al. (2017):

$$r = \sqrt{[t^2/(t^2 + d.f.)]} = \sqrt{F/(F + d.f.)} = \sqrt{(\chi^2/n)}$$

where t = t-statistics, F = F-statistics, *d.f.* = model denominator degrees of freedom, χ^2 = chi-squared statistics, and n = sample size. Effect sizes from inferential statistics were only retained when directions could be determined (e.g., F and χ^2 alone do not contain directional information).

Supplementary Tables

Supplementary Table S1 Trait categories associated with individual movement and the specific responses within these. Number of effect sizes, studies and species are shown. Responses with asterisks were corrected for direction. MR = metabolic rate.

Categorised trait	Specific response	Effect size (*k*)	Studies (*n*)	Species (*n*)
Cardiovascular	Haematocrit	4	2	2
Condition	Body condition	40	25	28
Condition	Glycogen content	2	1	1
Hormone	Glucocorticoids	7	6	5
Hormone	Testosterone	2	2	2
Immunity	White blood cell (WBC) count	2	1	1
Locomotor capacity	Cost of transport (CoT)*	8	2	1
Locomotor capacity	Endurance	14	7	7
Locomotor capacity	Max force	1	1	1
Locomotor capacity	Max jump	3	1	3
Locomotor capacity	Sprint speed	17	9	9
Locomotor capacity	Critical sustained swimming speed (U_{crit})	31	4	2
Metabolism	Aerobic scope	8	4	4
Metabolism	Basal MR	9	4	4
Metabolism	Field MR	4	3	4
Metabolism	Liver citrate synthase	1	1	1
Metabolism	Liver cytochrome c oxidase	1	1	1
Metabolism	Max MR	17	7	6
Metabolism	Muscle citrate synthase	13	2	2
Metabolism	Muscle cytochrome c oxidase	1	1	1
Metabolism	Muscle lactate dehydrogenase	12	1	1
Metabolism	Mitochondrial P/O ratio	2	1	1
Metabolism	Resting MR	32	22	20
Metabolism	Routine MR	25	11	10
Metabolism	Standard MR	11	8	8
Metabolism	Mitochondria State III respiration (State III)	2	1	1
Metabolism	Mitochondria State IV respiration (State IV)	2	1	1
Musculoskeletal	Muscle biomechanics	1	1	1

Supplementary Table S2 Trait categories associated with population range expansion and the specific responses within these. Number of effect sizes, studies and species are shown. Responses with asterisks were corrected for direction. MR = metabolic rate. MR = metabolic rate.

Category	Specific response	Effect size (*k*)	Studies (*n*)	Species (*n*)
Activity	Activity	64	11	6
Cardiovascular	Haematocrit	3	2	2
Cardiovascular	Haemoglobin	2	1	1
Cardiovascular	Heart mass	2	1	1
Cardiovascular	Red blood cell (RBC) count	1	1	1
Condition	Body condition	28	6	4
Condition	Fat score	1	1	1
Heat shock protein	Heat shock protein (HSP70)	3	1	1
Hormone	Corticosterone	20	7	4
Immunity	Bacterial killing assay (BKA)	6	3	2
Immunity	Neutrophil/lymphocyte ratio (NLR)	3	2	2
Immunity	White blood cell (WBC) count	1	1	1
Locomotor capacity	Endurance	38	9	7
Locomotor capacity	Sprint speed	30	3	1
Metabolic enzyme	Muscle citrate synthase	2	1	1
Metabolic enzyme	Muscle lactate dehydrogenase	2	1	1
Metabolism	Max MR	2	1	1
Metabolism	Resting MR	6	3	3
Metabolism	Routine MR	4	1	1
Metabolism	Standard MR	4	2	2
Musculoskeletal	Muscle profile	1	1	1
Musculoskeletal	Muscle ratio	3	1	1
Oxidative status	Reactive oxygen species	2	1	1
Oxidative status	Total antioxidant capacity	2	1	1
Thermal tolerance	Critical thermal maximum (*CT*max)	10	2	2
Thermal tolerance	Critical thermal minimum (*CT*min)*	12	6	4

Supplementary Table S3 Mean parameter estimates, estimate error, and 95% Bayesian credible intervals for the individual movement model, which includes three movement types (activity, exploration, dispersal), thermal strategy (ectotherm and endotherm), sex (female, male, mixed), age (juvenile and adult), origin (wild caught or lab reared) as fixed effects, and sampling error and publication year as covariates to account for sampling and publication bias. Random effects include the standard deviations (σ) for individual-level observations (effect size ID), study-level observations (study ID), species identity, and phylogenetic relatedness.

Parameters	Estimate	Est.Error	Q2.5	Q97.5
**Fixed effects**				
Activity	0.29	0.15	-0.003	0.58
Exploration	0.26	0.16	-0.05	0.57
Dispersal	0.35	0.18	-0.01	0.70
Thermal strategy	-0.08	0.11	-0.29	0.15
Male	-0.04	0.08	-0.19	0.12
Mixed	-0.01	0.10	-0.20	0.18
Age	0.09	0.10	-0.11	0.29
Origin	-0.10	0.09	-0.28	0.07
Sampling error	-0.51	0.35	-1.18	0.18
Publication year	-0.01	0.01	-0.02	0.002
**Random effects**				
σΔeffect size ID	0.4	0.02	0.36	0.45
σΔstudy ID	0.15	0.06	0.03	0.25
σΔspecies	0.08	0.06	0	0.21
σΔphylogeny	0.08	0.07	0	0.25

Supplementary Table S4 Heterogeneity of the individual movement analysis and population range expansion analysis. k = number of estimates, and I^2 = heterogeneity. Estimates shown correspond to modes and 95% highest posterior density intervals.

	**k (n)**	**I^2 [effect size] (%)**	**I^2 [study ID] (%)**	**I^2 [species] (%)**	**I^2 [phylogeny] (%)**	**I^2 [total] (%)**
Individual overall model	272	58.6 [40.7–72.8]	21.7 [4.3–33.6]	1.3 [0–23.3]	3.5 [0–26.0]	97.3 [96.6–98.0]
Population overall model	252	30.3 [19.2–38.7]	46.1 [32.6–60.7]	6.1 [0–23.3]	6.3 [0–33.0]	99.9 [99.9–100]

Supplementary Table S5 Mean parameter estimates, estimate error, and 95% credible intervals for the individual movement analysis with separate models for activity, exploration, and dispersal. Models include fixed-effect parameters for all extracted traits with >5 effect sizes, thermal strategy (ectotherm and endotherm), and sampling error with the publication year to account for sampling and publication bias. Random effects include the standard deviations (σ) for individual-level observations (effect size ID), study-level observations (study ID), species identity, and phylogenetic relatedness.

**Parameters - Activity only**	**Estimate**	**Est.Error**	**Q2.5**	**Q97.5**
**Fixed effects**				
Condition	0.31	0.22	-0.12	0.74
Locomotor capacity	0.38	0.19	-0.01	0.74
Metabolism	0.23	0.18	-0.15	0.58
Thermal strategy	-0.21	0.23	-0.67	0.23
Sampling error	-0.57	0.47	-1.49	0.37
Publication year	-0.01	0.01	-0.03	0.01
**Random effects**				
σΔeffect size ID	0.39	0.03	0.33	0.45
σΔstudy ID	0.28	0.07	0.15	0.42
σΔspecies	0.11	0.08	0	0.29
σΔphylogeny	0.15	0.14	0.01	0.53

**Parameters - Exploration only**	**Estimate**	**Est.Error**	**Q2.5**	**Q97.5**
**Fixed effects**				
Locomotor capacity	0.19	0.23	-0.24	0.66
Metabolism	0.12	0.21	-0.29	0.55
Thermal strategy	-0.03	0.22	-0.44	0.43
Sampling error	-0.59	0.86	-2.33	1.09
Publication year	0.00	0.03	-0.06	0.05
**Random effects**				
σΔeffect size ID	0.34	0.04	0.27	0.41
σΔstudy ID	0.11	0.08	0	0.31
σΔspecies	0.13	0.09	0.01	0.34
σΔphylogeny	0.16	0.15	0.01	0.55

**Parameters - Dispersal only**	**Estimate**	**Est.Error**	**Q2.5**	**Q97.5**
**Fixed effects**				
Condition	0.01	0.49	-0.99	0.97
Thermal strategy	0.28	0.45	-0.58	1.23
Sampling error	0.43	2.25	-4.14	4.93
Publication year	-0.04	0.03	-0.09	0.02
**Random effects**				
σΔeffect size ID	0.38	0.1	0.24	0.62
σΔstudy ID	0.21	0.16	0.01	0.61
σΔspecies	0.22	0.16	0.01	0.61
σΔphylogeny	0.37	0.33	0.01	1.2

Supplementary Table S6 Mean parameter estimates, estimate error, and 95% credible intervals for the individual movement analysis, grouped by metabolism, and locomotor performance traits, which includes fixed-effect parameters for all extracted responses with >5 effect sizes, age (juvenile, adult), thermal strategy (ectotherm and endotherm), and sampling error and publication year to account for sampling and publication bias. Random effects include the standard deviations (σ) for individual-level observations (effect size ID), study-level observations (study ID), species identity, and phylogenetic relatedness.

**Parameters - Metabolism**	**Estimate**	**Est.Error**	**Q2.5**	**Q97.5**
**Fixed effects**				
Aerobic scope	0.22	0.21	-0.2	0.63
Inactive MR	0.04	0.15	-0.25	0.34
Active MR	0.51	0.17	0.18	0.84
Metabolic enzyme	0.15	0.26	-0.37	0.66
Maximum MR	0.21	0.19	-0.15	0.57
Age	-0.07	0.13	-0.32	0.17
Sampling error	-0.79	0.52	-1.84	0.22
Publication year	-0.01	0.01	-0.02	0.01
**Random effects**				
σΔeffect size ID	0.38	0.03	0.33	0.44
σΔstudy ID	0.13	0.07	0.01	0.26
σΔspecies	0.09	0.07	0	0.25
σΔphylogeny	0.11	0.1	0	0.37

**Parameters - Locomotor cap.**	**Estimate**	**Est.Error**	**Q2.5**	**Q97.5**
**Fixed effects**				
CoT	-0.12	0.71	-1.55	1.3
Endurance	0.58	0.45	-0.3	1.51
Sprint speed	0.5	0.45	-0.33	1.46
U_{crit}	0.02	0.71	-1.4	1.47
Thermal strategy	-0.45	0.57	-1.61	0.68
Sampling error	0.02	1.39	-2.76	2.72
Publication year	0.01	0.02	-0.04	0.05
**Random effects**				
σΔeffect size ID	0.37	0.04	0.3	0.47
σΔstudy ID	0.24	0.16	0.01	0.61
σΔspecies	0.27	0.19	0.01	0.69
σΔphylogeny	0.44	0.33	0.02	1.27

Supplementary Table S7 Mean parameter estimates, estimate error, and 95% credible intervals for the individual movement analysis, with separate models for activity, exploration, dispersal. Models include fixed-effect parameters for all extracted taxa with >5 effect size, and sampling error with the publication year to account for sampling and publication bias. Random effects include the standard deviations (σ) for individual-level observations (effect size ID), study-level observations (study ID), and species identity.

**Parameters - Activity only**	**Estimate**	**Est.Error**	**Q2.5**	**Q97.5**
**Fixed effects**				
Amphibia	0.21	0.20	-0.18	0.61
Aves	0.08	0.26	-0.45	0.60
Invertebrate	0.37	0.17	0.04	0.70
Mammalia	0.11	0.19	-0.27	0.48
Osteichthyes	0.28	0.16	-0.03	0.60
Reptilia	0.24	0.30	-0.36	0.83
Sampling error	-0.59	0.51	-1.57	0.44
Publication year	-0.01	0.01	-0.03	0.01
Random effects				
σΔeffect size ID	0.4	0.03	0.35	0.46
σΔstudy ID	0.28	0.07	0.14	0.43
σΔspecies	0.11	0.08	0	0.29

**Parameters - Exploration only**	**Estimate**	**Est.Error**	**Q2.5**	**Q97.5**
**Fixed effects**				
Amphibia	0.08	0.24	-0.38	0.55
Aves	-0.03	0.20	-0.42	0.37
Invertebrate	0.09	0.20	-0.30	0.51
Mammalia	0.09	0.20	-0.30	0.48
Osteichthyes	0.17	0.24	-0.31	0.65
Reptilia	0.20	0.28	-0.35	0.76
Sampling error	-0.68	0.88	-2.45	1.02
Publication year	0.01	0.02	-0.04	0.06
**Random effects**				
σΔeffect size ID	0.33	0.03	0.27	0.4
σΔstudy ID	0.1	0.08	0	0.29
σΔspecies	0.13	0.09	0.01	0.35

**Parameters - Dispersal only**	**Estimate**	**Est.Error**	**Q2.5**	**Q97.5**
**Fixed effects**				
Amphibia	0.19	0.29	-0.39	0.78
Aves	0.35	0.33	-0.28	1.01
Invertebrate	-0.25	0.48	-1.19	0.72
Mammalia	0.19	0.27	-0.35	0.72
Sampling error	0.05	1.64	-3.24	3.27
Publication year	-0.04	0.01	-0.07	-0.01
**Random effects**				

σΔeffect size ID	0.37	0.07	0.25	0.54
σΔstudy ID	0.16	0.12	0.01	0.44
σΔspecies	0.18	0.13	0.01	0.48

Supplementary Table S8 Mean parameter estimates, estimate error and 95% credible intervals for the interaction between dispersal mode (aerial, aquatic, terrestrial) and temperature difference between range core and edge.

Parameters	Estimate	Est.Error	Q2.5	Q97.5
**Fixed effects**				
Intercept, β_0	3.51	0.09	3.33	3.69
Temperature difference	0.11	0.03	0.06	0.17
Diserpsal mode - Aquatic	-2.07	0.16	-2.39	-1.75
Diserpsal mode - Terrestrial	-0.54	0.14	-0.81	-0.27
Temperature difference x Diserpsal mode - Aquatic	0.81	0.21	0.40	1.23
Temperature difference x Diserpsal mode - Terrestrial	0.01	0.04	-0.07	0.09

Supplementary Table S9 Mean parameter estimates, estimate error and 95% credible intervals for the interaction between dispersal mode (aerial, aquatic, terrestrial) and precipitation (rainfall) difference between range core and edge.

Parameters	Estimate	Est.Error	Q2.5	Q97.5
**Fixed effects**				
Intercept, β_0	33.57	2.91	27.83	39.27
Rainfall difference	0.02	0.02	-0.01	0.05
Diserpsal mode - Aquatic	-13.37	4.18	-21.57	-5.12
Diserpsal mode - Terrestrial	-7.87	3.53	-14.80	-0.88
Rainfall difference x Diserpsal mode - Aquatic	-0.04	0.09	-0.22	0.13
Rainfall difference x Diserpsal mode - Terrestrial	-0.02	0.02	-0.05	0.01

Supplementary Table S10 Mean parameter estimates, estimate error, and 95% credible intervals for the interaction between dispersal mode (aerial, aquatic, terrestrial) and time since divergence between the core range and dispersal front. Random effects include the standard deviations (σ) for study-level observations (study ID), and species identity.

Parameters	Estimate	Est.Error	Q2.5	Q97.5
**Fixed effects**				
Intercept	-2.92	0.57	-4.04	-1.8
σ Intercept	-0.15	0.14	-0.41	0.14
Time	0.3	0.18	-0.06	0.65
Diserpsal mode - Aquatic	0.24	0.65	-1.05	1.5
Diserpsal mode - Terrestrial	-0.31	0.71	-1.71	1.1
Time x Diserpsal mode - Aquatic	0.01	0.28	-0.54	0.55
Time x Diserpsal mode - Terrestrial	0.08	0.2	-0.31	0.48
σ Time	0.07	0.04	-0.02	0.14
**Random effects**				
σΔstudy ID	0.86	0.16	0.58	1.21
σΔspecies	0.37	0.27	0.02	1

Supplementary Table S11 Mean parameter estimates, estimate error ,and 95% credible intervals for the population range expansion analysis, which includes the intercept, and sampling error with the publication year to account for sampling and publication bias. Random effects include the standard deviations (σ) for individual-level observations (effect size ID), study-level observations (study ID), species identity, and phylogenetic relatedness.

Parameters	Estimate	Est.Error	Q2.5	Q97.5
**Fixed effects**				
Intercept	0.25	0.15	-0.02	0.52
Sampling error	-0.27	0.17	-0.61	0.05
Publication year	0.01	0.02	-0.03	0.04
**Random effects**				
σΔeffect size ID	0.24	0.01	0.22	0.27
σΔstudy ID	0.39	0.06	0.29	0.52
σΔspecies	0.09	0.07	0	0.27
σΔphylogeny	0.13	0.12	0.01	0.42

Supplementary Table S12 Mean parameter estimates, estimate error, and 95% credible intervals for the population range expansion analysis, which includes fixed-effect parameters for all extracted traits with >5 effect sizes, sex, and sampling error with the publication year to account for sampling and publication bias. Random effects include the standard deviations (σ) for individual-level observations (effect size ID), study-level observations (study ID), species identity, and phylogenetic relatedness.

**Parameters**	**Estimate**	**Est.Error**	**Q2.5**	**Q97.5**
**Fixed effects**				
Activity	0.24	0.19	-0.14	0.65
Cardiovascular	0.10	0.21	-0.31	0.53
Condition	-0.09	0.24	-0.59	0.36
Hormone	0.60	0.23	0.16	1.05
Immunity	0.57	0.23	0.11	1.04
Locomotor capacity	0.25	0.19	-0.13	0.64
Metabolism	0.55	0.20	0.15	0.95
Thermal tolerance	0.33	0.20	-0.065	0.74
Male	-0.04	0.05	-0.15	0.06
Mixed	-0.19	0.08	-0.35	-0.03
Sampling error	-0.002	0.20	-0.39	0.37
Publication year	0.0009	0.02	-0.04	0.04
**Random effects**				
σΔeffect size ID	0.23	0.01	0.21	0.26
σΔstudy ID	0.45	0.07	0.33	0.6
σΔspecies	0.1	0.08	0	0.31
σΔphylogeny	0.16	0.14	0.01	0.51

Supplementary Table S13 Mean parameter estimates, estimate error, and 95% credible intervals for the population range expansion analysis, which includes fixed-effect parameters for all extracted taxa with >5 effect sizes, with sampling error and the publication year to account for sampling and publication bias. Random effects include the standard deviations (σ) for individual-level observations (effect size ID), study-level observations (study ID), and species identity.

**Parameters**	**Estimate**	**Est.Error**	**Q2.5**	**Q97.5**
**Fixed effects**				
Amphibia	0.29	0.14	0.002	0.57
Aves	0.53	0.21	0.10	0.94
Invertebrate	0.22	0.15	-0.07	0.51
Osteichthyes	0.23	0.23	-0.21	0.69
Male	-0.04	0.06	-0.15	0.07
Mixed	-0.15	0.08	-0.31	0.01
Sampling error	-0.13	0.20	-0.52	0.26
Publication year	0.01	0.02	-0.03	0.05
**Random effects**				
σΔeffect size ID	0.24	0.01	0.21	0.27
σΔstudy ID	0.39	0.06	0.29	0.52
σΔspecies	0.1	0.08	0	0.31

Supplementary Figures

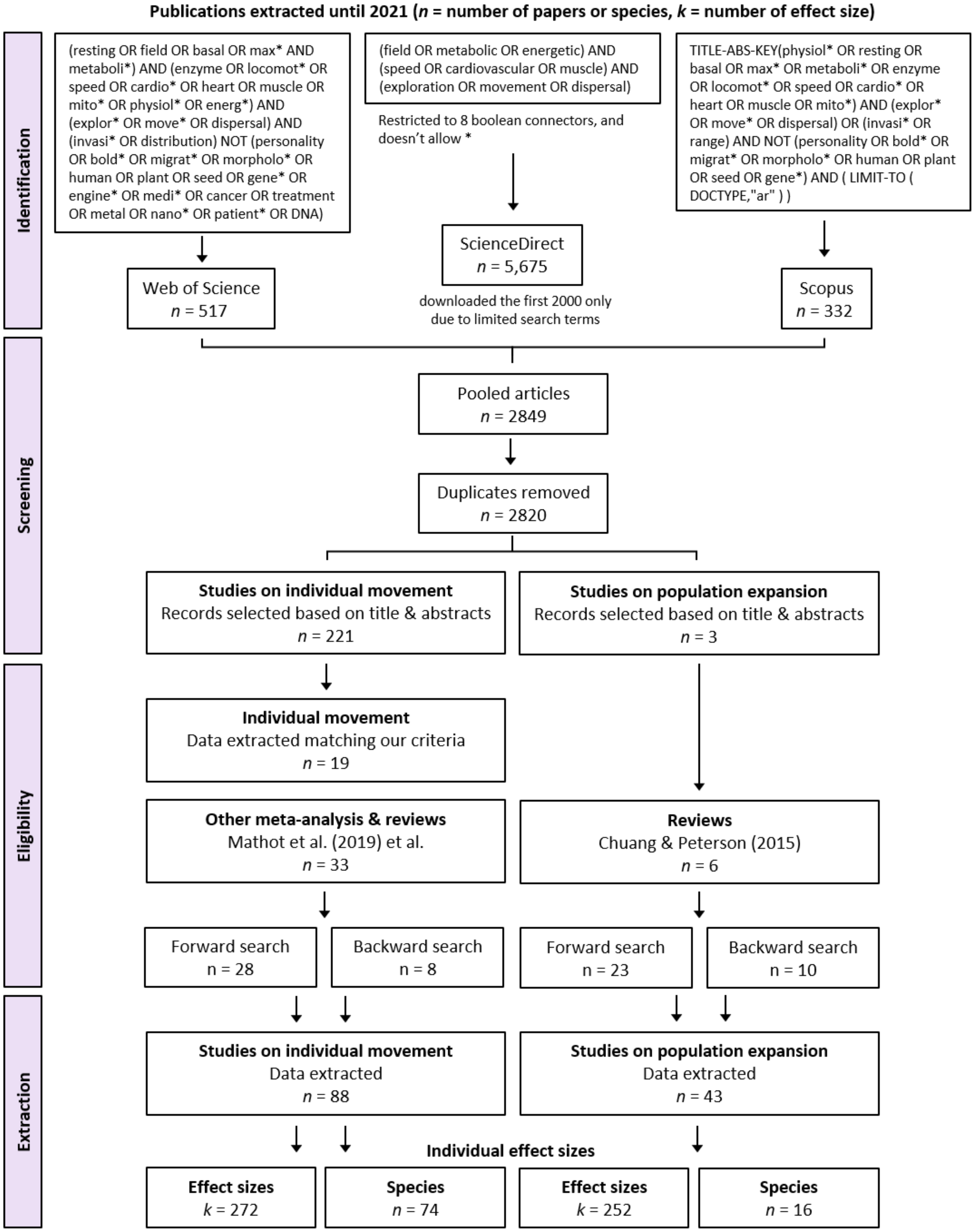

Supplementary Figure S1 PRISMA flow chart for the data-collection process. Search terms for each online database are shown. *n* = number of papers remaining after each stage of selection; *k* = number of effect sizes. Studies on individual movement include among-individual correlation of a physiological trait with activity, exploration, or dispersal. Studies on population expansion include comparison of physiology between population range core and edge.

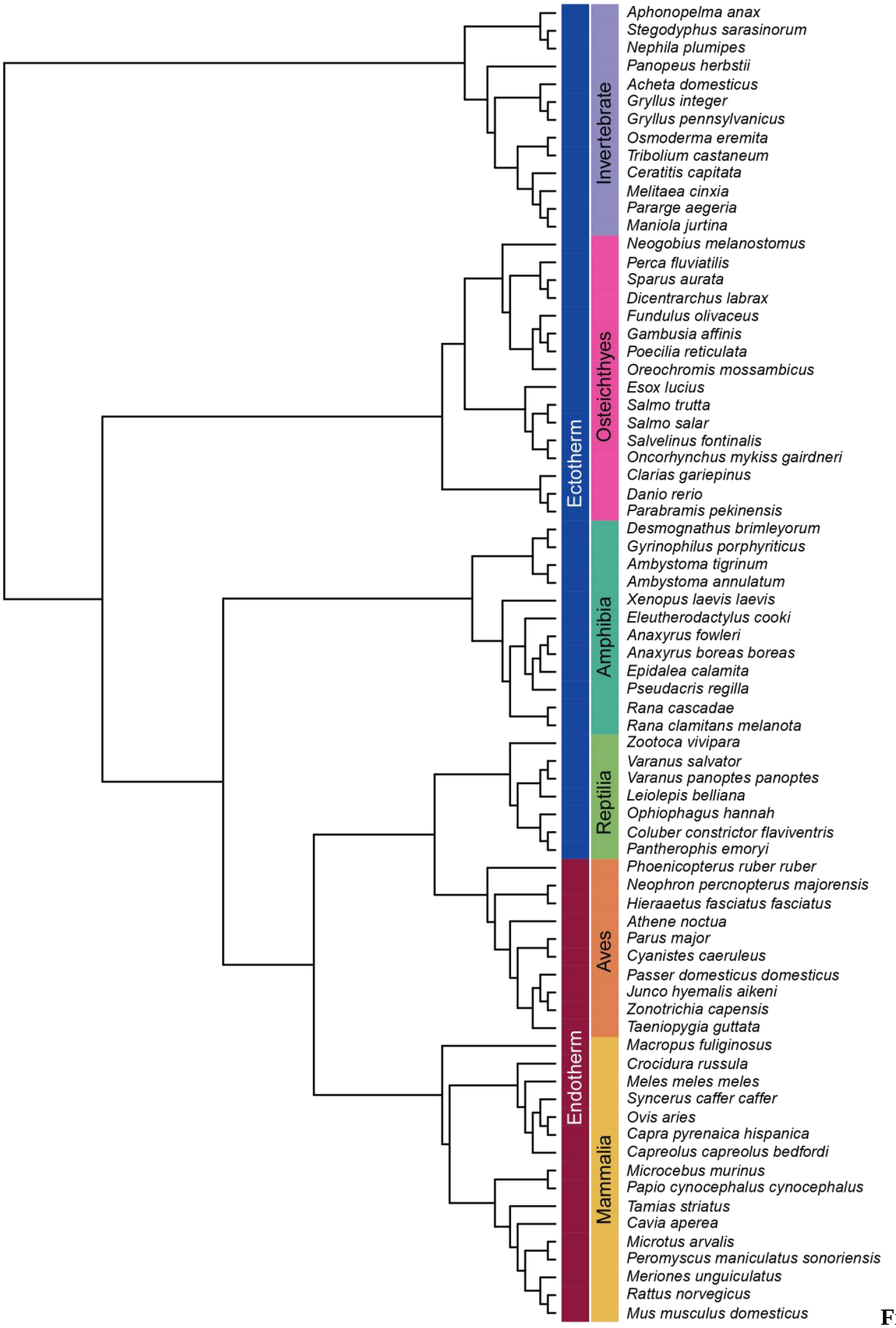

Fig

Supplementary Figure S2 Phylogenetic reconstruction for individual movement studies from the Open Tree of Life (https://tree.opentreeoflife.org/). The phylogeny was converted to a correlation matrix for the phylogenetic multilevel model.

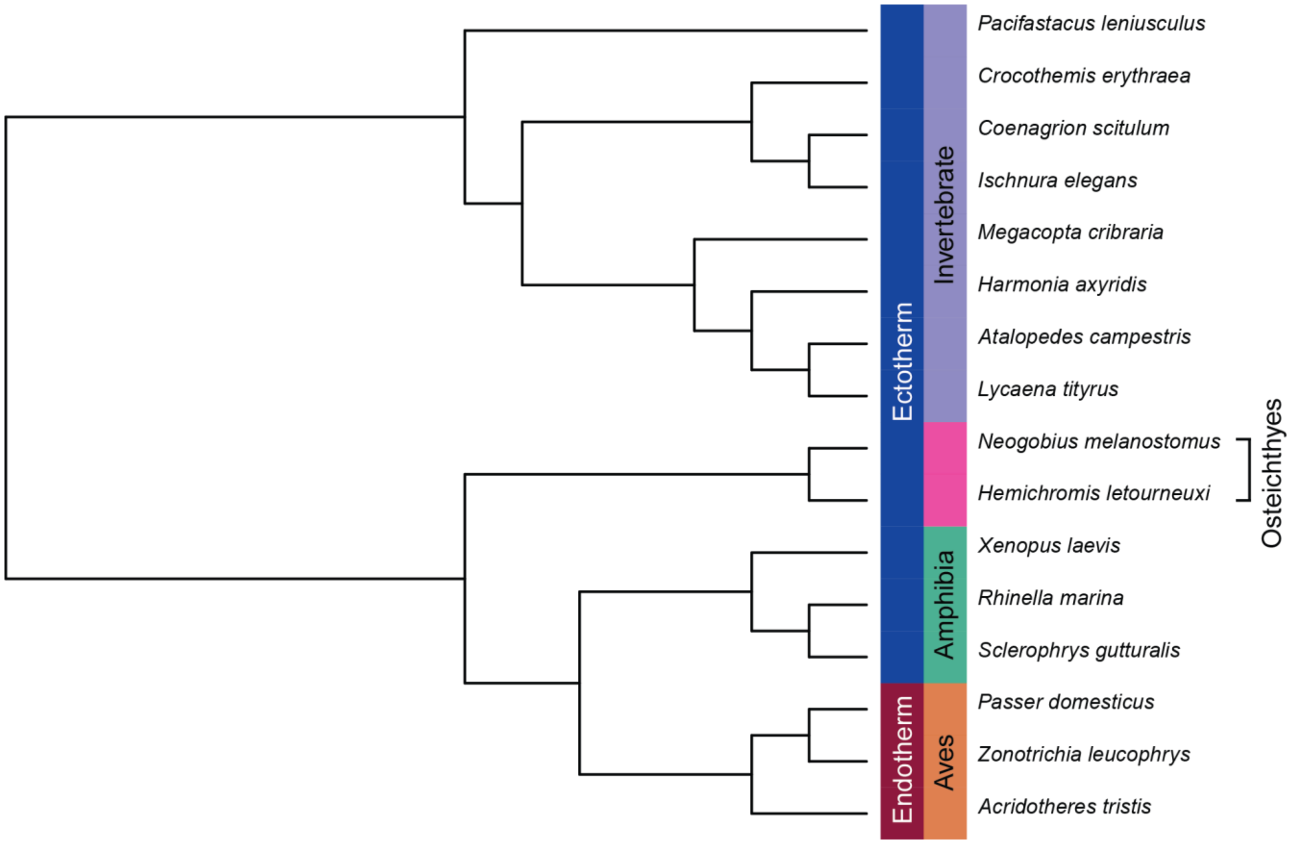

Supplementary Figure S3 Phylogenetic reconstruction for population range expansion studies from the Open Tree of Life (https://tree.opentreeoflife.org/). The phylogeny was converted to a correlation matrix for the phylogenetic multilevel model.

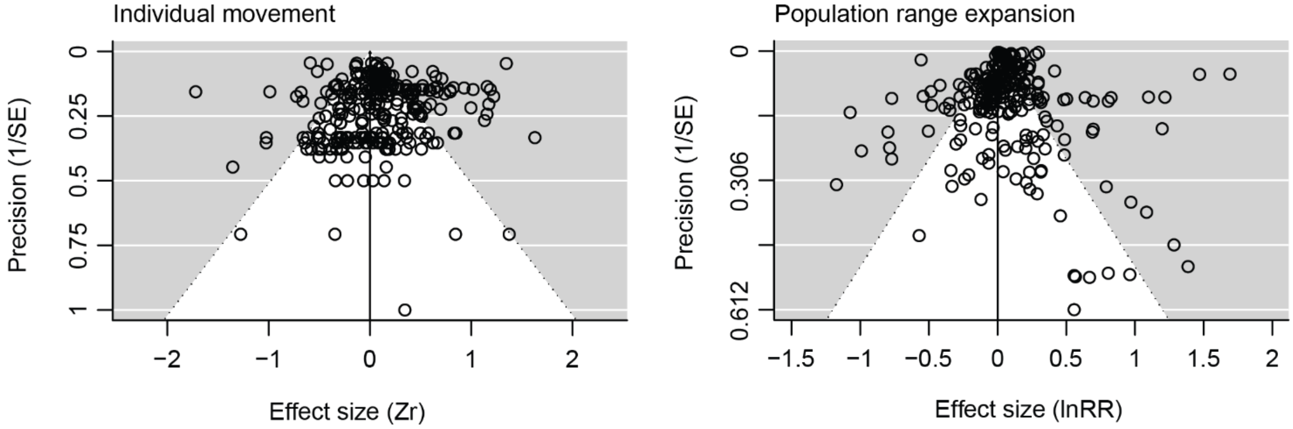

Supplementary Figure S4 Funnel plots for the individual movement data (left) and population range expansion data (right). The white area bordered by dashed lines represents the region of 95% pseudo confidence intervals where 95% of studies are expected to fall in the absence of bias and heterogeneity.

Supplementary references

Lipsey, M. W. and Wilson, D. B. (2001). Practical meta-analysis: SAGE publications, Inc.
Nakagawa, S. and Cuthill, I. C. (2007). Effect size, confidence interval and statistical significance: a practical guide for biologists. *Biol. Rev.* **82**, 591-605.
Nathan, R., Getz, W. M., Revilla, E., Holyoak, M., Kadmon, R., Saltz, D. and Smouse, P. E. (2008). A movement ecology paradigm for unifying organismal movement research. *Proc. Natl. Acad. Sci.* **105**, 19052-19059.
Noble, D. W., Lagisz, M., O'dea, R. E. and Nakagawa, S. (2017). Nonindependence and sensitivity analyses in ecological and evolutionary meta-analyses. *Mol. Ecol.* **26**, 2410-2425.
Zera, A. J. and Denno, R. F. (1997). Physiology and ecology of dispersal polymorphism in insects. *Annu. Rev. Entomol.* **42**, 207-230.

Studies included in the analysis

Individual movement

Addis, B. R., Tobalske, B. W., Davenport, J. M. and Lowe, W. H. (2019). A distance–performance trade-off in the phenotypic basis of dispersal. *Ecol. Evol.* **9**, 10644-10653.
Akinyi, M. Y., Gesquiere, L. R., Franz, M., Onyango, P. O., Altmann, J. and Alberts, S. C. (2017). Hormonal correlates of natal dispersal and rank attainment in wild male baboons. *Hormones and behavior* **94**, 153-161.
Arnold, P. A., Cassey, P. and White, C. R. (2017). Functional traits in red flour beetles: the dispersal phenotype is associated with leg length but not body size nor metabolic rate. *Funct. Ecol.* **31**, 653-661.
Atwell, J. W., Cardoso, G. C., Whittaker, D. J., Campbell-Nelson, S., Robertson, K. W. and Ketterson, E. D. (2012). Boldness behavior and stress physiology in a novel urban environment suggest rapid correlated evolutionary adaptation. *Behav. Ecol.* **23**, 960-969.
Auer, S. K., Salin, K., Anderson, G. J. and Metcalfe, N. B. (2016). Flexibility in metabolic rate and activity level determines individual variation in overwinter performance. *Oecologia* **182**, 703-712.
Azpillaga, M., Real, J. and Hernández-Matías, A. (2018). Effects of rearing conditions on natal dispersal processes in a long-lived predator bird. *Ecol. Evol.* **8**, 6682-6698.
Baktoft, H., Jacobsen, L., Skov, C., Koed, A., Jepsen, N., Berg, S., Boel, M., Aarestrup, K. and Svendsen, J. C. (2016). Phenotypic variation in metabolism and morphology correlating with animal swimming activity in the wild: relevance for the OCLTT (oxygen-and capacity-limitation of thermal tolerance), allocation and performance models. *Conserv. Physiol.* **4**, cov055.
Barbraud, C., Johnson, A. R. and Bertault, G. (2003). Phenotypic correlates of post-fledging dispersal in a population of greater flamingos: the importance of body condition. *J. Anim. Ecol.* **72**, 246-257.
Baugh, A. T., Davidson, S. C., Hau, M. and van Oers, K. (2017). Temporal dynamics of the HPA axis linked to exploratory behavior in a wild European songbird (*Parus major*). *General and comparative endocrinology* **250**, 104-112.
Baugh, A. T., Schaper, S. V., Hau, M., Cockrem, J. F., de Goede, P. and van Oers, K. (2012). Corticosterone responses differ between lines of great tits (*Parus major*) selected for divergent personalities. *General and Comparative Endocrinology* **175**, 488-494.
Belgrad, B. A., Karan, J. and Griffen, B. D. (2017). Individual personality associated with interactions between physiological condition and the environment. *Anim. Behav.* **123**, 277-284.
Biro, P. A., Thomas, F., Ujvari, B., Adriaenssens, B. and Beckmann, C. (2020). Spontaneous activity rates and resting metabolism: support for the allocation model of energy management at the among-individual level. *Ethology* **126**, 32-39.

Bouwhuis, S., Quinn, J. L., Sheldon, B. C. and Verhulst, S. (2014). Personality and basal metabolic rate in a wild bird population. *Oikos* **123**, 56-62.
Bredeweg, E. M., Morzillo, A. T., Thurman, L. L. and Garcia, T. S. (2019). The integrative effects of behavior and morphology on amphibian movement. *Ecol. Evol.* **9**, 1278-1288.
Careau, V., Buttemer, W. A. and Buchanan, K. L. (2014). Developmental stress can uncouple relationships between physiology and behaviour. *Biol. Lett.* **10**, 20140834.
Careau, V., Beauchamp, P. P., Bouchard, S. and Morand-Ferron, J. (2019). Energy metabolism and personality in wild-caught fall field crickets. *Physiol. Behav.* **199**, 173-181.
Careau, V., Thomas, D., Pelletier, F., Turki, L., Landry, F., Garant, D. and Réale, D. (2011). Genetic correlation between resting metabolic rate and exploratory behaviour in deer mice (*Peromyscus maniculatus*). *J. Evol. Biol.* **24**, 2153-2163.
Careau, V., Montiglio, P.-O., Garant, D., Pelletier, F., Speakman, J. R., Humphries, M. M. and Réale, D. (2015). Energy expenditure and personality in wild chipmunks. *Behav. Ecol. Sociobiol.* **69**, 653-661.
Chappell, M. A., Garland, T., Rezende, E. L. and Gomes, F. R. (2004). Voluntary running in deer mice: speed, distance, energy costs and temperature effects. *J. Exp. Biol.* **207**, 3839-3854.
Chappell, M. A., Garland, T., Robertson, G. F. and Saltzman, W. (2007). Relationships among running performance, aerobic physiology and organ mass in male Mongolian gerbils. *J. Exp. Biol.* **210**, 4179-4197.
Clark, S. R., Kreiser, B. R., Schaefer, J. F. and Stewart, L. K. (2019). Scale dependence of sex-specific movement in a small-bodied stream fish. *Freshw. Biol.* **64**, 1342-1353.
Crino, O., Buchanan, K. L., Trompf, L., Mainwaring, M. C. and Griffith, S. C. (2017). Stress reactivity, condition, and foraging behavior in zebra finches: effects on boldness, exploration, and sociality. *General and Comparative Endocrinology* **244**, 101-107.
Dubois, G. F., Le Gouar, P. J., Delettre, Y. R., Brustel, H. and Vernon, P. (2010). Sex-biased and body condition dependent dispersal capacity in the endangered saproxylic beetle *Osmoderma eremita* (Coleoptera: Cetoniidae). *J. Insect Conserv.* **14**, 679-687.
Farwell, M. and McLaughlin, R. L. (2009). Alternative foraging tactics and risk taking in brook charr (*Salvelinus fontinalis*). *Behav. Ecol.* **20**, 913-921.
Finkemeier, M. A., Trillmich, F. and Guenther, A. (2016). Match–mismatch experiments using photoperiod expose developmental plasticity of personality traits. *Ethology* **122**, 80-93.
Finstad, A., Einum, S., Forseth, T. and Ugedal, O. (2007). Shelter availability affects behaviour, size-dependent and mean growth of juvenile Atlantic salmon. *Freshw. Biol.* **52**, 1710-1718.
Fronhofer, E. A., Gut, S. and Altermatt, F. (2017). Evolution of density-dependent movement during experimental range expansions. *J. Evol. Biol.* **30**, 2165-2176.
Fu, C., Fu, S.-J., Wu, Q.-Y. and Cao, Z.-D. (2017). Predation threat modifies relationships between metabolism and behavioural traits but not their ecological relevance in Chinese bream. *Mar. Freshw. Behav. Physiol.* **50**, 329-344.
Garnier, A., Besnard, A., Crampe, J., Estèbe, J., Aulagnier, S. and Gonzalez, G. (2021). Intrinsic factors, release conditions and presence of conspecifics affect post-release dispersal after translocation of Iberian ibex. *Anim. Conserv.*
Gifford, M. E., Clay, T. A. and Careau, V. (2014). Individual (co) variation in standard metabolic rate, feeding rate, and exploratory behavior in wild-caught semiaquatic salamanders. *Physiol. Biochem. Zool.* **87**, 384-396.
Girardier, L., Clark, M. G. and Seydoux, J. (1995). Thermogenesis associated with spontaneous activity: an important component of thermoregulatory needs in rats. *J. Physiol.* **488**, 779-787.
Guenther, A. and Trillmich, F. (2015). Within-litter differences in personality and physiology relate to size differences among siblings in cavies. *Physiol. Behav.* **145**, 22-28.
Haag, C. R., Saastamoinen, M., Marden, J. H. and Hanski, I. (2005). A candidate locus for variation in dispersal rate in a butterfly metapopulation. *Proc. R. Soc. B. Biol. Sci.* **272**, 2449-2456.

Herrera, M., Castanheira, M. F., Conceição, L. E. and Martins, C. I. (2014). Linking risk taking and the behavioral and metabolic responses to confinement stress in gilthead seabream *Sparus aurata*. *Appl. Anim. Behav. Sci.* **155**, 101-108.
Hewison, A., Gaillard, J.-M., Morellet, N., Cagnacci, F., Debeffe, L., Cargnelutti, B., Gehr, B., Kröschel, M., Heurich, M. and Coulon, A. (2021). Sex differences in condition dependence of natal dispersal in a large herbivore: dispersal propensity and distance are decoupled. *Proc. R. Soc. B.* **288**, 20202947.
Hürlimann, M. L., Martin, J. G. and Bize, P. (2019). Evidence of phenotypic correlation between exploration activity and resting metabolic rate among populations across an elevation gradient in a small rodent species. *Behav. Ecol. Sociobiol.* **73**, 1-10.
Jahn, M. and Seebacher, F. (2019). Cost of transport is a repeatable trait but is not determined by mitochondrial efficiency in zebrafish (*Danio rerio*). *J. Exp. Biol.* **222**.
Kasumovic, M. and Seebacher, F. (2018). Casual movement speed but not maximal locomotor capacity predicts mate searching success. *J. Evol. Biol.* **31**, 438-445.
Killen, S. S., Marras, S. and McKenzie, D. J. (2011). Fuel, fasting, fear: routine metabolic rate and food deprivation exert synergistic effects on risk-taking in individual juvenile European sea bass. *J. Anim. Ecol.* **80**, 1024-1033.
Killen, S. S., Marras, S., Ryan, M. R., Domenici, P. and McKenzie, D. J. (2012). A relationship between metabolic rate and risk-taking behaviour is revealed during hypoxia in juvenile European sea bass. *Funct. Ecol.* **26**, 134-143.
Kluen, E., Siitari, H. and Brommer, J. E. (2014). Testing for between individual correlations of personality and physiological traits in a wild bird. *Behav. Ecol. Sociobiol.* **68**, 205-213.
Klug, P. E., Fill, J. and With, K. A. (2011). Spatial ecology of eastern yellow-bellied racer (*Coluber constrictor flaviventris*) and Great Plains rat snake (*Pantherophis emoryi*) in a contiguous tallgrass-prairie landscape. *Herpetologica* **67**, 428-439.
Krams, I. A., Niemelä, P. T., Trakimas, G., Krams, R., Burghardt, G. M., Krama, T., Kuusik, A., Mänd, M., Rantala, M. J. and Mänd, R. (2017). Metabolic rate associates with, but does not generate covariation between, behaviours in western stutter-trilling crickets, *Gryllus integer*. *Proc. R. Soc. B. Biol. Sci.* **284**, 20162481.
Laskowski, K., Monk, C., Polverino, G., Alós, J., Nakayama, S., Staaks, G., Mehner, T. and Arlinghaus, R. (2016). Behaviour in a standardized assay, but not metabolic or growth rate, predicts behavioural variation in an adult aquatic top predator *Esox lucius* in the wild. *J. Fish Biol.* **88**, 1544-1563.
Le Galliard, J. F., Paquet, M., Cisel, M. and Montes-Poloni, L. (2013). Personality and the pace-of-life syndrome: variation and selection on exploration, metabolism and locomotor performances. *Funct. Ecol.* **27**, 136-144.
Le Roy, A. and Seebacher, F. (2018). Transgenerational effects and acclimation affect dispersal in guppies. *Funct. Ecol.* **32**, 1819-1831.
Lebeau, J., Wesselingh, R. A. and Van Dyck, H. (2016). Floral resource limitation severely reduces butterfly survival, condition and flight activity in simplified agricultural landscapes. *Oecologia* **180**, 421-427.
Lei, J., Booth, D. T. and Dwyer, R. G. (2017). Spatial ecology of yellow-spotted goannas adjacent to a sea turtle nesting beach. *Aust. J. Zool.* **65**, 77-86.
Lei, J., Booth, D. T., Rusli, M. U. and Zhang, Z. (2020). Spatial Ecology of Asian Water Monitors Adjacent to a Sea Turtle Nesting Beach. *Zoological Science* **38**.
Lei, J., Binti Yusof, N. S., Wu, N. C., Zhang, Z. and Booth, D. T. (2021). The burrowing ecology of a tropical lizard (*Leiolepis belliana*). *Herpetologica* **77**, 37-44.
Liebl, A. L. and Martin, L. B. (2012). Exploratory behaviour and stressor hyper-responsiveness facilitate range expansion of an introduced songbird. *Proc. R. Soc. B. Biol. Sci.* **279**, 4375-4381.
Louppe, V., Courant, J., Videlier, M. and Herrel, A. (2018). Differences in standard metabolic rate at the range edge versus the center of an expanding invasive population of *Xenopus laevis* in the West of France. *J. Zool.* **305**, 163-172.

Lowe, W. H., Likens, G. E. and Cosentino, B. J. (2006). Self-organisation in streams: The relationship between movement behaviour and body condition in a headwater salamander. *Freshw. Biol.* **51**, 2052-2062.
Maes, J., Van Damme, R. and Matthysen, E. (2013). Individual and among-population variation in dispersal-related traits in Natterjack toads. *Behav. Ecol.* **24**, 521-531.
Maldonado, K., Van Dongen, W. F., Vásquez, R. A. and Sabat, P. (2012). Geographic variation in the association between exploratory behavior and physiology in rufous-collared sparrows. *Physiol. Biochem. Zool.* **85**, 618-624.
Marshall, B. M., Strine, C. T., Jones, M. D., Artchawakom, T., Silva, I., Suwanwaree, P. and Goode, M. (2019). Space fit for a king: spatial ecology of king cobras (*Ophiophagus hannah*) in Sakaerat Biosphere Reserve, Northeastern Thailand. *Amphib-Reptilia* **40**, 163-178.
McDonald, D., Keeler, R. and McFarlane, W. (2007). The relationships among sprint performance, voluntary swimming activity, and social dominance in juvenile rainbow trout. *Physiol. Biochem. Zool.* **80**, 619-634.
McKenzie, D. J., Belão, T. C., Killen, S. S., Blasco, F. R., Svendsen, M. B. and Rantin, F. T. (2021). Evidence that standard metabolic rate and risk-taking to breathe air are linked to boldness, activity level and exploratory behaviour in a catfish. *bioRxiv*.
Meylan, S., Belliure, J., Clobert, J. and de Fraipont, M. (2002). Stress and body condition as prenatal and postnatal determinants of dispersal in the common lizard (*Lacerta vivipara*). *Hormones and Behavior* **42**, 319-326.
Moore, M. P. and Whiteman, H. H. (2016). Natal philopatry varies with larval condition in salamanders. *Behav. Ecol. Sociobiol.* **70**, 1247-1255.
Munn, A. J., Kalkman, L., Skeers, P., Roberts, J., Bailey, J. and Dawson, T. J. (2016). Field metabolic rate, movement distance, and grazing pressures by western grey kangaroos (*Macropus fuliginosus melanops*) and Merino sheep (*Ovis aries*) in semi-arid Australia. *Mamm. Biol.* **81**, 423-430.
Newar, S. L. and Careau, V. (2018). The fast and the curious: locomotor performance and exploratory behaviour in eastern chipmunks. *Behav. Ecol. Sociobiol.* **72**, 1-12.
Niitepõld, K., Mattila, A. L., Harrison, P. J. and Hanski, I. (2011). Flight metabolic rate has contrasting effects on dispersal in the two sexes of the Glanville fritillary butterfly. *Oecologia* **165**, 847-854.
Niitepõld, K., Smith, A. D., Osborne, J. L., Reynolds, D. R., Carreck, N. L., Martin, A. P., Marden, J. H., Ovaskainen, O. and Hanski, I. (2009). Flight metabolic rate and Pgi genotype influence butterfly dispersal rate in the field. *Ecology* **90**, 2223-2232.
Oliveira, F. G., Mathias, M. d. L., Rychlik, L., Tapisso, J. T. and von Merten, S. (2020). Metabolic and behavioral adaptations of greater white-toothed shrews to urban conditions. *Behav. Ecol.* **31**, 1334-1343.
Ousterhout, B. H. and Semlitsch, R. D. (2018). Effects of conditionally expressed phenotypes and environment on amphibian dispersal in nature. *Oikos* **127**, 1142-1151.
Peña-Villalobos, I., Casanova-Maldonado, I., Lois, P., Palma, V. and Sabat, P. (2020). Costs of exploratory behavior: the energy trade-off hypothesis and the allocation model tested under caloric restriction. *Sci. Rep.* **10**, 1-13.
Pennekamp, F., Clobert, J. and Schtickzelle, N. (2019). The interplay between movement, morphology and dispersal in *Tetrahymena* ciliates. *PeerJ* **7**, e8197.
Plummer, M. V. and Congdon, J. D. (1996). Rates of metabolism and water flux in free-ranging racers, *Coluber constrictor*. *Copeia*, 8-14.
Rezende, E. L., Gomes, F. R., Chappell, M. A. and Garland Jr, T. (2009). Running behavior and its energy cost in mice selectively bred for high voluntary locomotor activity. *Physiol. Biochem. Zool.* **82**, 662-679.
Rogowitz, G. L. and Sánchez-Rivoleda, J. (1999). Locomotor performance and aerobic capacity of the cave coqui, *Eleutherodactylus cooki*. *Copeia*, 40-48.

Ros, A. F., Becker, K. and Oliveira, R. F. (2006). Aggressive behaviour and energy metabolism in a cichlid fish, *Oreochromis mossambicus*. *Physiol. Behav.* **89**, 164-170.
Royauté, R., Greenlee, K., Baldwin, M. and Dochtermann, N. A. (2015). Behaviour, metabolism and size: phenotypic modularity or integration in *Acheta domesticus*? *Anim. Behav.* **110**, 163-169.
Schliehe-Diecks, S. (2012). Sex-specific differences in dispersal propensities and their consequences for grey mouse lemurs (*Microcebus murinus*): Niedersächsische Staats-und Universitätsbibliothek Göttingen.
Searcy, C. A., Gilbert, B., Krkošek, M., Rowe, L. and McCauley, S. J. (2018). Positive correlation between dispersal and body size in green frogs (*Rana clamitans*) naturally colonizing an experimental landscape. *Can. J. Zool.* **96**, 1378-1384.
Seebacher, F. and Kazerouni-Ghanizadeh, E. (2021). Water flow and temperature interact to determine oxidative status, swimming performance, and dispersal of mosquitofish (*Gambusia holbrooki*). *Freshw. Biol.*
Seebacher, F., Little, A. G. and James, R. S. (2015). Skeletal muscle contractile function predicts activity and behaviour in zebrafish. *J. Exp. Biol.* **218**, 3878-3884.
Serrano, D., Cortés-Avizanda, A., Zuberogoitia, I., Blanco, G., Benítez, J. R., Ponchon, C., Grande, J. M., Ceballos, O., Morant, J. and Arrondo, E. (2021). Phenotypic and environmental correlates of natal dispersal in a long-lived territorial vulture. *Sci. Rep.* **11**, 1-13.
Spaan, R. S., Epps, C. W., Ezenwa, V. O. and Jolles, A. E. (2019). Why did the buffalo cross the park? Resource shortages, but not infections, drive dispersal in female African buffalo (*Syncerus caffer*). *Ecol. Evol.* **9**, 5651-5663.
Steyn, V. M., Mitchell, K. A. and Terblanche, J. S. (2016). Dispersal propensity, but not flight performance, explains variation in dispersal ability. *Proc. R. Soc. B. Biol. Sci.* **283**, 20160905.
Stoltey, T. and Shillington, C. (2009). Metabolic rates and movements of the male tarantula *Aphonopelma anax* during the mating season. *Can. J. Zool.* **87**, 1210-1220.
Thorlacius, M., Hellström, G. and Brodin, T. (2015). Behavioral dependent dispersal in the invasive round goby *Neogobius melanostomus* depends on population age. *Curr. Zool.* **61**, 529-542.
Toscano, B. J. and Monaco, C. J. (2015). Testing for relationships between individual crab behavior and metabolic rate across ecological contexts. *Behav. Ecol. Sociobiol.* **69**, 1343-1351.
Vaanholt, L. M., Magee, V. and Speakman, J. R. (2012). Factors predicting individual variability in diet-induced weight loss in MF1 mice. *Obesity* **20**, 285-294.
Van Dyck, H. and Holveck, M.-J. (2016). Ecotypic differentiation matters for latitudinal variation in energy metabolism and flight performance in a butterfly under climate change. *Sci. Rep.* **6**, 1-9.
Walton, M. (1988). Relationships among metabolic, locomotory, and field measures of organismal performance in the Fowler's toad (*Bufo woodhousei fowleri*). *Physiol. Zool.* **61**, 107-118.
Woodroffe, R., Macdonald, D. and Da Silva, J. (1995). Dispersal and philopatry in the European badger, *Meles meles*. *J. Zool.* **237**, 227-239.

Population range expansion

Assis, V. R., Gardner, S. T., Smith, K. M., Gomes, F. R. and Mendonça, M. T. (2020). Stress and immunity: Field comparisons among populations of invasive cane toads in Florida. *J. Exp. Zool. Part A* **333**, 779-791.
Barsotti, A. M. G., Madelaire, C. B., Wagener, C., Titon Jr, B., Measey, J. and Gomes, F. R. (2021). Challenges of a novel range: Water balance, stress, and immunity in an invasive toad. *Comp. Biochem. Physiol. Part A Mol. Integr. Physiol.* **253**, 110870.
Brandner, J., Cerwenka, A. F., Schliewen, U. K. and Geist, J. (2013). Bigger is better: characteristics of round gobies forming an invasion front in the Danube River. *PLoS One* **8**, e73036.
Brandner, J., Cerwenka, A. F., Schliewen, U. K. and Geist, J. (2018). Invasion strategies in round goby (*Neogobius melanostomus*): Is bigger really better? *PloS one* **13**, e0190777.

Brusch, G. A., Christian, K., Brown, G. P., Shine, R. and DeNardo, D. F. (2019). Cane toads (*Rhinella marina*) rely on water access, not drought tolerance, to invade xeric Australian environments. *Oecologia* **189**, 307-316.
Burstal, J., Clulow, S., Colyvas, K., Kark, S. and Griffin, A. S. (2020). Radiotracking invasive spread: Are common mynas more active and exploratory on the invasion front? *Biol. Invasions* **22**, 2525-2543.
Carbonell, J. A. and Stoks, R. (2020). Thermal evolution of life history and heat tolerance during range expansions toward warmer and cooler regions. *Ecology* **101**, e03134.
Carbonell, J. A., Wang, Y.-J. and Stoks, R. (2021). Evolution of cold tolerance and thermal plasticity in life history, behaviour and physiology during a poleward range expansion. *J. Anim. Ecol.*
Courant, J., Secondi, J., Guillemet, L., Vollette, E. and Herrel, A. (2019). Rapid changes in dispersal on a small spatial scale at the range edge of an expanding population. *Evol. Ecol.* **33**, 599-612.
Crozier, L. (2003). Winter warming facilitates range expansion: cold tolerance of the butterfly *Atalopedes campestris*. *Oecologia* **135**, 648-656.
Gruber, J., Brown, G., Whiting, M. J. and Shine, R. (2017). Geographic divergence in dispersal-related behaviour in cane toads from range-front versus range-core populations in Australia. *Behav. Ecol. Sociobiol.* **71**, 38.
Hirsch, P. E., Thorlacius, M., Brodin, T. and Burkhardt-Holm, P. (2017). An approach to incorporate individual personality in modeling fish dispersal across in-stream barriers. *Ecol. Evol.* **7**, 720-732.
Hudina, S., Žganec, K. and Hock, K. (2015). Differences in aggressive behaviour along the expanding range of an invasive crayfish: an important component of invasion dynamics. *Biol. Invasions* **17**, 3101-3112.
Hudson, C. M., Vidal-García, M., Murray, T. G. and Shine, R. (2020). The accelerating anuran: evolution of locomotor performance in cane toads (*Rhinella marina*, Bufonidae) at an invasion front. *Proc. R. Soc. B.* **287**, 20201964.
Kosmala, G., Christian, K., Brown, G. and Shine, R. (2017). Locomotor performance of cane toads differs between native-range and invasive populations. *Roy. Soc. Open Sci.* **4**, 170517.
Kosmala, G. K., Brown, G. P. and Shine, R. (2020). Laid-back invaders: Cane toads (*Rhinella marina*) down-regulate their stress responses as they colonize a harsh climate. *Global Ecology and Conservation* **24**, e01248.
Krause, J. S., Chmura, H. E., Pérez, J. H., Quach, L. N., Asmus, A., Word, K. R., McGuigan, M. A., Sweet, S. K., Meddle, S. L. and Gough, L. (2016). Breeding on the leading edge of a northward range expansion: differences in morphology and the stress response in the arctic Gambel's white-crowned sparrow. *Oecologia* **180**, 33-44.
Liebl, A. L. and Martin, L. B. (2012). Exploratory behaviour and stressor hyper-responsiveness facilitate range expansion of an introduced songbird. *Proc. R. Soc. B. Biol. Sci.* **279**, 4375-4381.
Llewellyn, D., Thompson, M. B., Brown, G. P., Phillips, B. L. and Shine, R. (2012). Reduced investment in immune function in invasion-front populations of the cane toad (*Rhinella marina*) in Australia. *Biol. Invasions* **14**, 999-1008.
Llewelyn, J., Phillips, B. L., Alford, R. A., Schwarzkopf, L. and Shine, R. (2010). Locomotor performance in an invasive species: cane toads from the invasion front have greater endurance, but not speed, compared to conspecifics from a long-colonised area. *Oecologia* **162**, 343-348.
Lombaert, E., Estoup, A., Facon, B., Joubard, B., Grégoire, J. C., Jannin, A., Blin, A. and Guillemaud, T. (2014). Rapid increase in dispersal during range expansion in the invasive ladybird *Harmonia axyridis*. *J. Evol. Biol.* **27**, 508-517.
Lopez, D., Jungman, A. and Rehage, J. (2012). Nonnative African jewelfish are more fit but not bolder at the invasion front: a trait comparison across an Everglades range expansion. *Biol. Invasions* **14**, 2159-2174.

Louppe, V., Courant, J. and Herrel, A. (2017). Differences in mobility at the range edge of an expanding invasive population of *Xenopus laevis* in the west of France. *J. Exp. Biol.* **220**, 278-283.
Louppe, V., Courant, J., Videlier, M. and Herrel, A. (2018). Differences in standard metabolic rate at the range edge versus the center of an expanding invasive population of *Xenopus laevis* in the West of France. *J. Zool.* **305**, 163-172.
Madelaire, C. B., Barsotti, A. M., Wagener, C., Sugano, Y. Y. V., Baxter-Gilbert, J., Gomes, F. R. and Measey, J. (2020). Challenges of dehydration result in a behavioral shift in invasive toads. *Behav. Ecol. Sociobiol.* **74**, 1-15.
Martin, L. B. and Liebl, A. L. (2014). Physiological flexibility in an avian range expansion. *General and Comparative endocrinology* **206**, 227-234.
Martin, L. B., Kilvitis, H. J., Thiam, M. and Ardia, D. R. (2017a). Corticosterone regulation in house sparrows invading Senegal. *General and comparative endocrinology* **250**, 15-20.
Martin, L. B., Kilvitis, H. J., Brace, A. J., Cooper, L., Haussmann, M. F., Mutati, A., Fasanello, V., O'Brien, S. and Ardia, D. R. (2017b). Costs of immunity and their role in the range expansion of the house sparrow in Kenya. *J. Exp. Biol.* **220**, 2228-2235.
McCann, S. M., Kosmala, G. K., Greenlees, M. J. and Shine, R. (2018). Physiological plasticity in a successful invader: rapid acclimation to cold occurs only in cool-climate populations of cane toads (*Rhinella marina*). *Conserv. Physiol.* **6**, cox072.
Merwin, A. C. (2019). Flight capacity increases then declines from the core to the margins of an invasive species' range. *Biol. Lett.* **15**, 20190496.
Mittan, C. S. and Zamudio, K. R. (2019). Rapid adaptation to cold in the invasive cane toad *Rhinella marina*. *Conserv. Physiol.* **7**, coy075.
Myles-Gonzalez, E., Burness, G., Yavno, S., Rooke, A. and Fox, M. G. (2015). To boldly go where no goby has gone before: boldness, dispersal tendency, and metabolism at the invasion front. *Behav. Ecol.* **26**, 1083-1090.
Padilla, P., Courant, J. and Herrel, A. (2019). Allocation trade-offs impact organ size and muscle architecture in an invasive population of *Xenopus laevis* in Western France. *J. Anat.* **235**, 1057-1064.
Phillips, B. L., Brown, G. P. and Shine, R. (2010). Evolutionarily accelerated invasions: the rate of dispersal evolves upwards during the range advance of cane toads. *J. Evol. Biol.* **23**, 2595-2601.
Pizzatto, L., Both, C., Brown, G. and Shine, R. (2017). The accelerating invasion: dispersal rates of cane toads at an invasion front compared to an already-colonized location. *Evol. Ecol.* **31**, 533-545.
Raffard, A., Therry, L., Finn, F., Koch, K., Brodin, T., Blanchet, S. and Cote, J. (2020). Does range expansion modify trait covariation? A study of a northward expanding dragonfly. *Oecologia* **192**, 565-575.
Rebrina, F., Skejo, J., Lucić, A. and Hudina, S. (2015). Trait variability of the signal crayfish (*Pacifastacus leniusculus*) in a recently invaded region reflects potential benefits and trade-offs during dispersal. *Aquat. Invasions* **10**, 41-50.
Reim, E., Blesinger, S., Förster, L. and Fischer, K. (2018). Successful despite poor flight performance: range expansion is associated with enhanced exploratory behaviour and fast development. *J. Evol. Biol.* **31**, 1165-1179.
Selechnik, D., West, A. J., Brown, G. P., Fanson, K. V., Addison, B., Rollins, L. A. and Shine, R. (2017). Effects of invasion history on physiological responses to immune system activation in invasive Australian cane toads. *PeerJ* **5**, e3856.
Stuart, K. C., Shine, R. and Brown, G. P. (2019). Proximate mechanisms underlying the rapid modification of phenotypic traits in cane toads (*Rhinella marina*) across their invasive range within Australia. *Biol. J. Linn. Soc.* **126**, 68-79.
Therry, L., Zawal, A., Bonte, D. and Stoks, R. (2014). What factors shape female phenotypes of a poleward-moving damselfly at the edge of its range? *Biol. J. Linn. Soc.* **112**, 556-568.

Tracy, C. R., Christian, K. A., Baldwin, J. and Phillips, B. L. (2011). Cane toads lack physiological enhancements for dispersal at the invasive front in Northern Australia. *Biol. Open* **1**, 37-42.
Vimercati, G., Davies, S. J. and Measey, J. (2018). Rapid adaptive response to a mediterranean environment reduces phenotypic mismatch in a recent amphibian invader. *J. Exp. Biol.* **221**, jeb174797.
